# Mapping of quantitative trait loci for early seed germination using whole-genome resequencing chromosome segment substitution lines of aus Kasalath in the background of japonica Nipponbare

**DOI:** 10.3389/fpls.2025.1691426

**Published:** 2025-10-20

**Authors:** Siyao Shan, Muhan Ye, Jue Ruan, Sheng Teng, Min Yu

**Affiliations:** ^1^ College of Plant Science and Technology, Huazhong Agricultural University, Wuhan, China; ^2^ Guangdong Laboratory of Lingnan Modern Agriculture, Agricultural Genomics Institute at Shenzhen, Chinese Academy of Agricultural Sciences, Shenzhen, China; ^3^ International Research Center for Environmental Membrane Biology and Department of Horticulture, Foshan University, Foshan, China; ^4^ Key Laboratory of Microbiological Metrology, Measurement and Bio-product Quality Security, State Administration for Market Regulation, College of Life Sciences, China Jiliang University, Hangzhou, China

**Keywords:** rice, seed germination, seed vigor, chromosome segment substitution lines (CSSL), whole-genome resequencing

## Abstract

Direct-seeding systems in rice require cultivars with enhanced seed vigor to ensure rapid and uniform germination. In this study, a population of 42 chromosome segment substitution lines (CSSLs) derived from aus Kasalath in the japonica Nipponbare background, coupled with whole-genome resequencing data, was analyzed to identify quantitative trait loci (QTLs) associated with early seed germination. Phenotypic traits, including germination percentage (GP), germination rate (GR), and germination index (GI), were measured over two consecutive years to ensure robustness. Six stable QTLs were identified, with *qSV*-*1* on chromosome 1 (30.10–30.94 Mb) emerging as the most consistent across evaluations for GP and GI. Functional annotation and gene expression analyses of the 840-kb *qSV*-*1* interval pinpointed *C3H10* and *OsOFP3* as the primary candidate genes for further investigation. This study underscores the effectiveness of whole-genome resequencing paired with the CSSL platform for precise QTL identification, thereby providing critical genetic resources for improving seed vigor through marker-assisted breeding and functional genomics.

## Introduction

Rice (*Oryza sativa* L.) is a staple crop pivotal to global food security, feeding over half the world’s population, particularly in Asia (Seck et al., 2012). The increasing adoption of direct-seeding systems, driven by advances in irrigation methods, herbicide efficiency, and labor cost reductions, has emphasized the need for rice varieties with enhanced seed vigor. Seed vigor ensures rapid and uniform germination, forming the foundation of strong seedling establishment, critical aspects for the efficiency of direct-seeding methods (Mahender et al., 2015; Hafeez-ur-Rehman et al., 2019). Enhanced seed vigor benefits field emergence, suppresses weed competition, and stabilizes yield under diverse environmental stresses such as drought, salinity, and extreme temperature fluctuations (Cui et al., 2002; Hafeez-ur-Rehman et al., 2019). Consequently, advancing our understanding of the genetic and molecular mechanisms underlying seed germination vigor is imperative for developing rice varieties tailored for direct-seeding systems.

Seed germination begins with water uptake (imbibition), followed by radicle protrusion through the seed coat—a transition from dormancy to active growth (Nonogaki et al., 2010). This intricate trait is governed by physiological and biochemical processes, including carbohydrate mobilization, hormone signaling, and redox homeostasis (Wang et al., 2025b; Xu et al., 2025). Among phytohormones, gibberellic acid (GA) and abscisic acid (ABA) exhibit opposing roles: GA activates hydrolytic enzymes like α-amylase to mobilize stored starch in the endosperm, while ABA inhibits premature germination, ensuring dormancy maintenance (Chen et al., 2020). Other hormones modulate seed germination by interacting with the GA and ABA pathways (Shu et al., 2016; Penfield, 2017). Additionally, reactive oxygen species (ROS) facilitate cell expansion and signaling during imbibition, contributing to seed germination initiation (Bailly, 2019; Wang et al., 2025b). Identifying functional genes that regulate these processes is key to improving rice seed vigor for direct-seeding systems.

Understanding the genetic basis of seed vigor has seen significant advancements through quantitative trait locus (QTL) mapping and genome-wide association studies (GWASs). These methods have identified multiple QTLs linked to germination speed, vigor, and stress tolerance during germination; however, challenges persist due to the complexity of germination traits and the low resolution of traditional genetic markers (Cui et al., 2002; Wang et al., 2011; Sasaki et al., 2013; Jin et al., 2018; Dai et al., 2022; Xu et al., 2023). For example, He et al. (2022) identified seven QTLs regulating seed germination vigor under optimal conditions but successfully cloned only one causal gene, *OsHIPL1*, for the major QTL *qSV3*, demonstrating its role in modulating ABA levels and *OsABI* expression (He et al., 2022). Similarly, GWASs and linkage mapping unveiled over 30 QTLs associated with low-temperature germination (LTG), yet most causal genes remain uncharacterized (Jiang et al., 2020). Notable exceptions include *qLTG3*-*1*, which promotes germination under cold conditions, and *OsSAP16*, which mediates cold-stress tolerance during germination (Fujino et al., 2008; Wang et al., 2018). Under salinity stress, Wang et al. fine-mapped 16 QTLs, identifying *qSE3* as mediated by *OsHAK21*, a potassium transporter crucial for germination and seedling establishment (Wang et al., 2011; He et al., 2019). Additionally, five QTLs with underlying tolerance to flooding during germination were identified, such as *OsTPP7*, which enhances trehalose metabolism under submergence conditions (Wang et al., 2023). Despite progress, the fine mapping and cloning of causal genes for germination traits remain limited, underscoring the need for precise genetic characterization techniques.

Chromosome segment substitution lines (CSSLs) provide a powerful platform for high-resolution QTL mapping due to their ability to reduce genetic background noise and facilitate the detection of minor-effect loci. CSSLs, compared to traditional populations like F2 or recombinant inbred lines (RILs), enable detailed examination of traits influenced by environmental factors (Yamamoto et al., 2009). Kasalath, a genetically diverse aus-type indica rice variety, is frequently used as a donor parent for CSSL development. It is known for superior agronomic traits such as seed dormancy, mesocotyl elongation, and seed longevity (Miura et al., 2002; Ballini et al., 2013; Liu et al., 2015; Sasaki et al., 2015; Fu et al., 2019). Kasalath has contributed to the identification of dormancy-related QTLs. Notably, Sugimoto et al. (2010) cloned *Sdr4*, which regulates dormancy through ABA signaling (Sugimoto et al., 2010). Similarly, *SD6* is a bHLH transcription factor controlling dormancy by regulating ABA biosynthesis (*NCED*) and catabolism (*ABA8OX3*) in response to temperature changes (Xu et al., 2022). These findings highlight the precision of CSSLs in mapping and cloning genes controlling complex traits like seed vigor.

Integrating whole-genome resequencing with CSSL populations enhances QTL mapping by supplying detailed information about substituted chromosomal regions (Xu et al., 2010; Balakrishnan et al., 2019). This study utilized CSSLs derived from aus Kasalath in the japonica Nipponbare background, along with high-throughput resequencing, to investigate early seed germination. Through a high-quality SNP (Single Nucleotide Polymorphism)-based genetic map constructed from deep resequencing data, six QTLs were identified, including *qSV*-*1*, the most significant locus, which was fine-mapped to an 80-kb interval. The combined use of CSSL mapping and resequencing paves the way for identifying novel genetic determinants of seed vigor, contributing to the development of high-performing rice varieties adapted to direct-seeding systems.

## Materials and methods

### Plant materials and growth conditions

A population of 42 CSSLs, with japonica Nipponbare as the recurrent parent and aus Kasalath as the donor parent, was used. Developed by Dr. M. Yano (National Institute of Agrobiological Sciences, Japan), each CSSL carries one or a few Kasalath-derived chromosome segments in the Nipponbare background.

The CSSLs and parental lines were cultivated at the Shenzhen Agricultural Genomics Institute, China (22°35′N, 114°29′E), during July 2021 (summer) and April 2022 (spring). Lines were planted in a block consisting of four rows with six plants per row, spaced 50 cm × 35 cm. Mature seeds were harvested at physiological maturity, air-dried to approximately 12% moisture content, and stored at room temperature until germination assays.

### Seed germination assays and trait measurements

Mature, plump seeds from each CSSL and parental line were selected for germination experiments. Seed dormancy was broken by incubating seeds at 45°C for 7 days (Sugimoto et al., 2010). For each line, 50 seeds per replicate (three biological replicates) were placed in 9-cm Petri dishes lined with two layers of Whatman filter paper moistened with 30 mL distilled water. Germination tests were conducted in a growth chamber at 30°C ± 1°C, 70% relative humidity, and a 12-h light/12-h dark photoperiod with a light intensity of 200 μmol·m^−2^·s^−1^ (white fluorescent light).

Germination was recorded daily from days 2 to 7 post-imbibition, with germination defined as radicle protrusion ≥1 mm. Three seed vigor indicators were calculated: germination percentage (GP) = (number of germinated seeds within 7 days/total seeds) × 100%. Germination rate (GR) = (number of germinated seeds within 3 days/total seeds) × 100%. Germination index (GI) = Σ (Gt/Dt), where Gt is the number of seeds germinated on day Dt, and Dt is the corresponding day (Wang et al., 2010). Phenotypic data (GP, GR, and GI) were collected in 2021 and 2022 to account for environmental variation.

### DNA extraction for next-generation sequencing

Genomic DNA was extracted from 2-cm leaf segments of 30-day-old seedlings using the cetyltrimethylammonium bromide (CTAB) method (Rogers and Bendich). Leaf samples were ground in liquid nitrogen, incubated in CTAB buffer at 65°C for 30 minutes, and purified with chloroform:isoamyl alcohol (24:1). DNA was precipitated with isopropanol, washed with 70% ethanol, and resuspended in TE buffer. Quality was assessed using a NanoDrop 2000 spectrophotometer (*Thermo Fisher Scienti, USA*) and 1% agarose gel electrophoresis, ensuring ≥50 ng/µL and an A260/A280 ratio of 1.8–2.0.

### High-throughput genotyping and substituted segment analysis

Whole-genome resequencing of the 42 CSSLs and parental lines was performed by Beijing Novogene Technology Co., Ltd., using the Illumina HiSeq Xten platform (HiSeq PE150 strategy, approximately 10× coverage). Genomic DNA (≥1 µg per sample) was sheared, and paired-end libraries were constructed. The *O. sativa* cv. Nipponbare IRGSP 1.0 reference genome was used for alignment (Kawahara et al., 2013), and SNPs were called using SAMtools v1.9 (Li et al., 2009) with a minimum read depth of 5 and mapping quality of 30. Substituted segments were identified by comparing SNP profiles between CSSLs and Nipponbare. Crossovers were identified between adjacent blocks with different genotypes, and bins were defined as intervals between crossovers (Zhou et al., 2015). Physical maps and segment lengths were constructed using IRGSP 1.0 coordinates.

### CSSL-based QTL mapping

QTLs were mapped by comparing GR and GI of each CSSL with those of Nipponbare using single-factor ANOVA (p < 0.05). CSSLs with significantly higher GR or GI were considered to carry vigor-related QTLs, confirmed by F-tests (p < 0.05). Only QTLs detected in both years were retained. Overlapping substituted segments with significant phenotypic differences were identified as QTL intervals, while non-significant overlaps were excluded (Wang et al., 2021). QTL boundaries were refined using SNP data.

## Results

### Kasalath exhibits stronger germination vigor than Nipponbare

**Figure 1:**
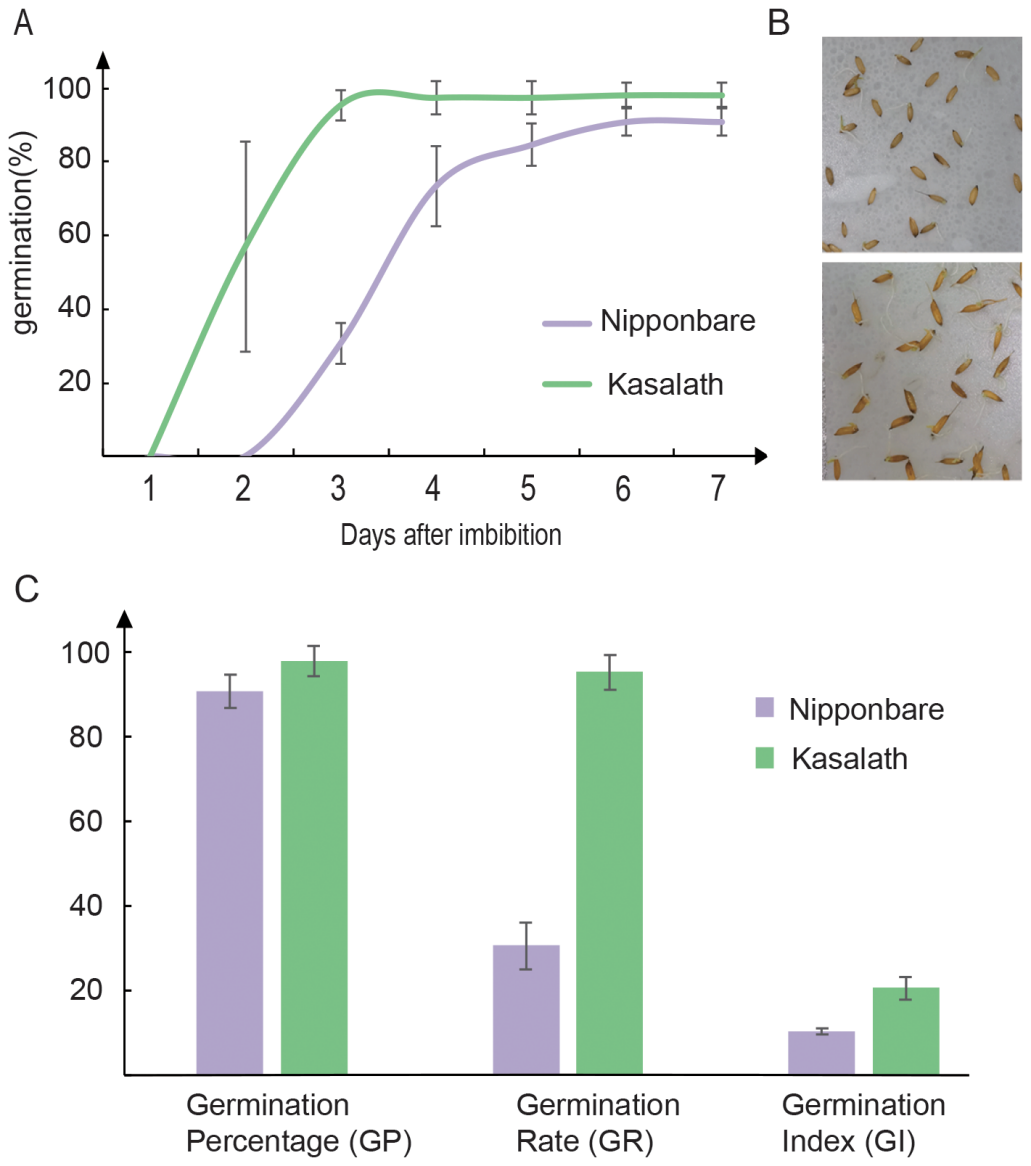
Comparison of seed germination vigor between aus Kasalath and japonica Nipponbare. **(A)** Germination dynamics of Kasalath and Nipponbare over 7 days after imbibition. Seeds were after-ripened, and dormancy was broken by incubation at 45°C for 7 days prior to germination tests. Germination (%) was defined as radicle protrusion ≥1 mm. **(B)** The germination phenotype of Nipponbare (top) and Kasalath (bottom) after 3 days of imbibition. **(C)** Comparison of germination percentage (GP), germination rate (GR), and germination index (GI) between the two varieties. Data are presented as means ± SE of three biological replicates, each with 50 seeds. Kasalath exhibited significantly higher GR and GI than Nipponbare.

After breaking seed dormancy (45°C for 7 days), both Kasalath and Nipponbare exhibited high germination percentages (>90%), indicating excellent seed quality (**Figure 1**). However, the GR of Kasalath was significantly higher than that of Nipponbare. After 3 days of imbibition, Kasalath achieved a GR exceeding 95%, while Nipponbare showed a substantially lower GR of approximately 30%. Statistical analysis confirmed Kasalath’s germination rate (95.33% ± 4.16%) as significantly higher than that of Nipponbare (30.72% ± 5.55%).

The superiority of Kasalath’s seed vigor was further supported by GI analysis. Kasalath’s GI value (20.63 ± 2.74) was markedly higher than Nipponbare’s (10.38 ± 0.75) (**Figure 1**). These results affirm Kasalath’s strong seed vigor, identifying it as a suitable donor parent for mapping QTLs related to early seed germination.

### High-throughput genotyping of CSSLs

**Figure 2:**
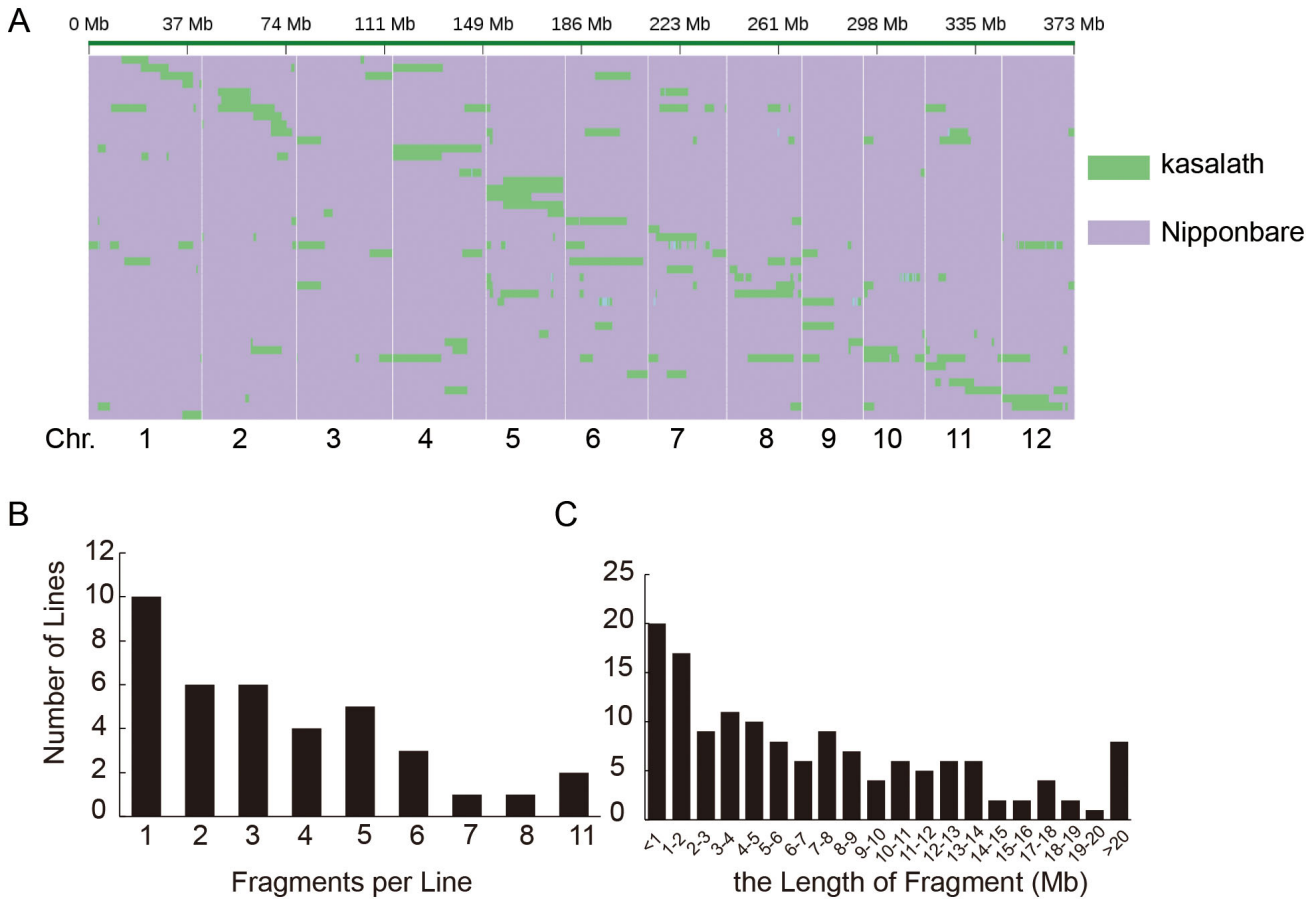
High-throughput genotyping of chromosome segment substitution lines (CSSLs). **(A)** Physical maps of substituted chromosome segments in CSSLs. Green represents Kasalath-derived segments, and purple represents Nipponbare background. The x-axis shows chromosomes (Chrs. 1–12) and their physical positions (in Mb). **(B)** Distribution of the number of substituted fragments per CSSL line. The x-axis indicates the number of fragments per line, and the y-axis shows the number of CSSL lines. **(C)** Distribution of the length of substituted fragments (in Mb). The x-axis represents the length of fragments, and the y-axis shows the number of fragments.

High-throughput whole-genome resequencing of 42 CSSLs resulted in 444.1 Gb of clean paired-end sequencing data (approximately 9.25 Gb per line, equivalent to 10× genome coverage). Using filtered SNP positions, physical maps of substituted chromosome segments were successfully constructed (**Figure 2A**).

A total of 141 substituted Kasalath-derived segments were mapped across the CSSLs (**Table 1**). The average number of substituted segments ranged from 1 to 11 per CSSL line, with approximately 12 segments per chromosome on average (**Figure 2B**). The distribution of substituted segments varied, ranging from 7 on chromosome 3 to 19 on chromosome 5.

**Table 1:** Summary of substituted chromosome segments in CSSLs.

Chromosome	Number of substituted segments	Total length of substituted segments (Mb)	Coverage rate (%)
1	17	35.21	81.36
2	14	26.82	74.65
3	7	25.25	69.35
4	9	35.50	100.00
5	19	29.50	98.47
6	11	31.25	100.00
7	14	22.55	75.92
8	13	25.60	90.00
9	8	17.78	77.50
10	8	22.16	95.49
11	12	21.17	72.94
12	9	27.23	98.92
total	141	320.01	85.76

CSSLs, chromosome segment substitution lines.

Segment length analysis revealed that 45 segments were shorter than 2 Mb, while 37 segments exceeded 10 Mb in length (**Figure 2C**). The total length of substituted regions across a genome was approximately 972.3 Mb (**Supplementary Table 1**), averaging 6.75 Mb per segment. Substituted regions collectively covered 320.01 Mb, with chromosomal coverage rates varying from 69.35% on chromosome 3 to 100% on chromosomes 4 and 6 (**Table 1**).

### Phenotypic variation of seed vigor traits

**Figure 3:**
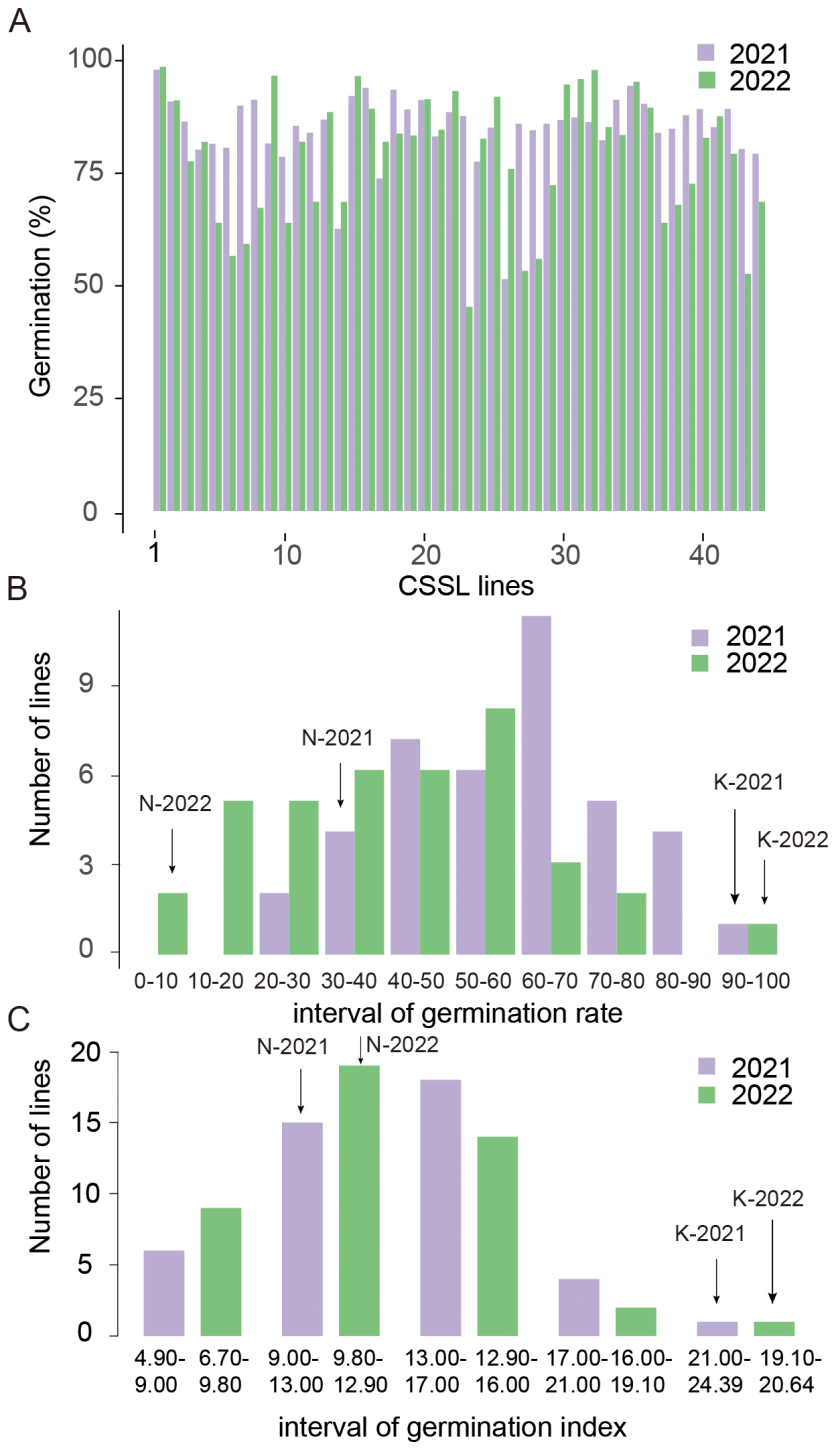
Phenotypic variation of seed vigor traits. **(A)** Germination percentage of CSSL lines across two years (2021, orange; 2022, blue). The vertical axis shows germination percentage, and the horizontal axis lists CSSL lines. **(B)** Distribution of CSSL lines by germination rate (GR) intervals across two years. The horizontal axis represents GR intervals (%), and the vertical axis shows the number of lines. “N-2021/N-2022” marks Nipponbare’s GR ranges in respective years, and “K-2021/K-2022” marks Kasalath’s. **(C)** Distribution of CSSL lines by germination index (GI) intervals across two years. The horizontal axis shows GI intervals, and the vertical axis shows the number of lines. “N-2021/N-2022” and “K-2021/K-2022” mark parent ranges.

The phenotypic variations in seed vigor traits, including seed germination percentage, germination speed, and germination index, were observed among the CSSLs and their parent lines for QTL analysis. No significant differences in germination percentage were observed between Nipponbare (91.06%) and Kasalath (98.3%) for their after-ripened seeds. Most CSSLs exhibited germination percentages ranging from 70% to 98%, although a few lines displayed percentages as low as 50% in one of the two years, potentially due to environmental factors affecting growth in that specific year (**Figure 3A**; **Supplementary Table 1**).

Correlation analysis revealed strong positive relationships between GR and GI, with an average correlation coefficient of 0.9 over two years of analysis. In contrast, GP exhibited weaker correlations with GR and GI, ranging from 0.6 to 0.8, indicating that GI serves as a reliable indicator for evaluating seed vigor (**Supplementary Table 2**).

The CSSL population showed significant divergence for germination rate and germination index traits. The value of GR ranged from 5.33% to 98.64%. The value of GI ranged from 6.71 to 24.39 (**Figures 3B, C**; **Supplementary Table 1**). Continuous and normally distributed phenotypic patterns occurred for both traits across two years, reflecting their quantitative inheritance and making them suitable for QTL analysis. Additionally, transgressive segregation was observed, with some CSSLs exhibiting values beyond those of both parents, suggesting that seed vigor traits are controlled by multiple genes.

### QTL mapping for seed vigor traits

Resequencing-based physical maps of the CSSLs were converted into bin maps for QTL analysis. Based on phenotypic data collected over two years, six QTLs associated with seed vigor were identified, located on chromosomes 1, 3, 4, 8, 9, and 11. The phenotypic variance explained by a single QTL ranged from 7.5% to 15.2%. Among these, the major QTL, *qSV-1*, exhibited the highest LOD (Logarithm of Odds) score (6.4). The additive effect of *qSV* showed a positive indication that the positive allele from Kasalath contributed to the early seed germination (**Table 2**).

**Table 2:** QTLs associated with seed vigor traits in CSSLs.

QTL	Chr	Location	LOD	Phenotypic variance explained (%)	Add	Co-location germination treated gene
*qSV-1*	1	30.09–30.93	6.4	15.2	1.28	Unknown
*qSV-3*	3	24.49–25.90	5.2	12.9	1.22	*OsNCED3*
*qSV-4*	4	19.95–22.96	4.1	10.8	1.96	*OsABA1*
*qSV-8*	8	0.98–2.90	4.8	9.8	0.77	Unknown, next to LOL1
*qSV-9*	9	22.75–23.01	3.4	7.5	2.31	*OsCNL2*
*qSV-11*	11	18.68–19.92	5.8	13.1	1.45	Unknown

QTLs, quantitative trait loci; CSSLs, chromosome segment substitution lines.

Of the six QTLs, three had overlap with previously reported genes, while the remaining three represented novel findings. For the previously reported QTLs, *qSV*-*3*, mapped between 24.49 and 25.90 Mb on chromosome 3, contains the gene *OsNCED3*. The overexpression of *OsNCED3* enhances resistance to pre-harvest sprouting (PHS) and increases grain size and weight by optimally regulating the ABA/GA ratio in the embryo (Chen et al., 2023). Similarly, *qSV*-*4*, located between 19.95 and 22.96 Mb on chromosome 4, incorporates the gene *OsABA1*, which synthesizes ABA to inhibit early seed germination (Agrawal et al., 2001). Meanwhile, *qSV*-*9*, positioned between 22.75 and 23.01 Mb on chromosome 9, harbors the gene *OsCNL2*, a pivotal enzyme in salicylic acid (SA) biosynthesis. *OsCNL2* promotes germination under submerged conditions via OsGH3-mediated indole-acetic acid catabolism (Wang et al., 2024b).

The three novel uncovered QTLs, with *qSV*-*8* identified between 0.98 and 2.90 Mb on chromosome 8, lie near *OsLOL1* (approximately 3.46 Mb), which facilitates seed germination by modulating gibberellin biosynthesis through interaction with OsbZIP58 (Wu et al., 2014). The remaining loci, *qSV*-*1* and *qSV*-*11*, lacked known germination-related genes, although *qSV*-*1* was delimited to a highly promising interval of 830 kb. These findings underscore the reliability of the identified QTLs and highlight the dominant influence of the Kasalath allele on early germination behaviors.

### Validation of the candidates of *qSV*-*1*

**Figure 4:**
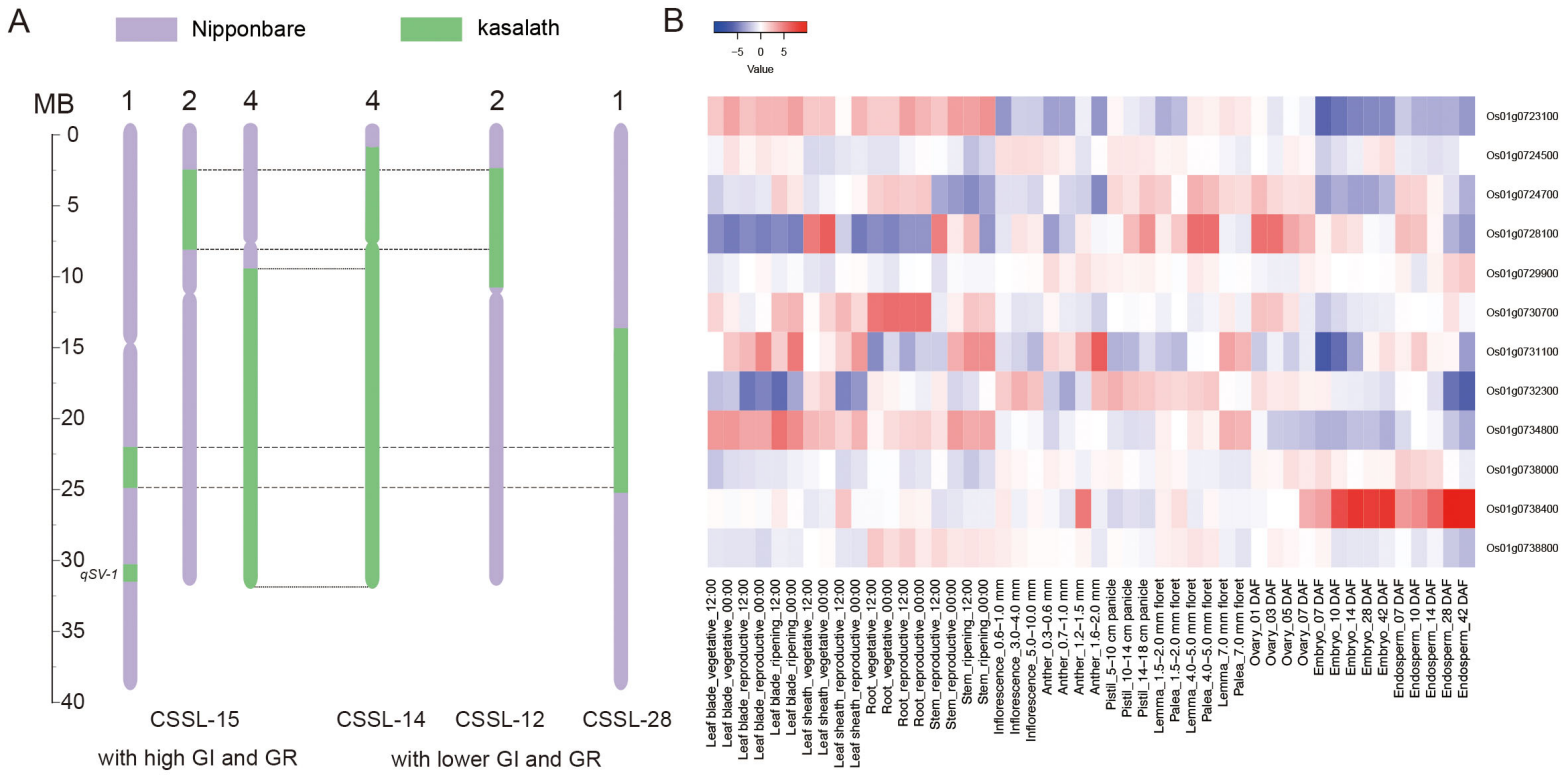
Validation of candidates for qSV-1. **(A)** Genomic segment comparison of chromosome segment substitution lines (CSSLs) to refine the qSV-1 locus. Purple represents the Nipponbare background, and green represents Kasalath-derived segments. CSSL-15 [with high germination index (GI) and germination rate (GR)] and CSSL-12, CSSL-14, and CSSL-28 (with lower GI and GR) are shown. By comparing overlapping regions, the qSV-1 locus is delimited to an 830-kb interval (30.09–30.93 Mb on chromosome 1). **(B)** Heatmap of gene expression patterns for annotated genes within the qSV-1 interval. The color scale (blue to red) represents expression values. Rows list genes; columns represent different rice tissues/stages.

CSSL-15 (containing *qSV*-*1*) exhibited consistently high GR and GI during two years of experiments (**Figure 4A**; **Supplementary Table 1**). Despite overlap in genomic regions of CSSL-15, CSSL-12, CSSL-14, and CSSL-28, the last three CSSL lines showed lower GR and GI. Comparison of overlapping regions further delimited the qSV-1 locus to an 830-kb region spanning 30.09–30.93 Mb on chromosome 1.

From this interval, 20 annotated genes were identified based on the Rice Genome Annotation Project (http://rice.plantbiology.msu.edu/) (**Supplementary Table 3**). Expression data from RiceXPro revealed potential candidates such as Os01g0738400 (C3H10), which shows seed-specific expression during embryo and endosperm development and responds to stress conditions like drought, high salinity, and ABA. Another candidate (Seong et al., 2020), Os01g0732300 (OsOFP3), functions as a repressor of brassinosteroid signaling and shows altered expression patterns during seed development (Xiao et al., 2020). Sequence analysis revealed five SNPs in the promoter region (1.0 kb upstream of the start codon) and multiple SNPs 1–2 kb upstream of the start codon for *C3H10*, which may influence its expression across genetic backgrounds. Additionally, a lysine-to-arginine amino acid change, located outside of the CCCH motif, was identified in the coding region, potentially affecting protein stability or protein–protein interactions. For *OsOFP3*, an ATCC deletion in its promoter region and a 3-bp indel in the coding region, resulting in the deletion of a proline residue in Kasalath, may disrupt its protein–protein interaction ability and affect its role in seed development and germination (**Supplementary File 1**). These preliminary findings suggest that some genes within this interval may play critical roles in regulating seed germination traits, warranting further validation through functional studies.

## Discussion

Our study aimed to improve seed vigor in rice varieties optimized for direct-seeding systems, a critical attribute for enhancing productivity and reducing labor costs in modern rice cultivation. Through a population of 42 CSSLs derived from aus Kasalath in the japonica Nipponbare genetic background, coupled with whole-genome resequencing, six QTLs regulating early seed germination were identified. Among these, *qSV*-1 emerged as the most prominent and consistent locus. Fine mapping delimited its interval to an 80.5-kb region and revealed potential candidate genes, including *C3H10* and *OsOFP3*. These findings provide strategic genetic resources for marker-assisted breeding and deepen our understanding of molecular pathways underlying seed vigor traits in rice.

Seed vigor is a highly complex trait influenced by multiple genetic determinants and environmental factors (Reed et al., 2022). Aus rice varieties are renowned for their high genetic diversity and adaptability to stress-prone environments, including drought, low soil fertility, and fluctuating climatic conditions (Liao et al., 2022; Sar et al., 2024). Moreover, aus rice exhibits superior early seedling vigor, making it an invaluable genetic resource for studying and improving seed germination-related traits (Basha et al., 2024). Our findings confirm that aus-derived CSSLs outperform japonica rice in seed vigor traits, showing continuous phenotypic segregation in seed vigor indicators such as GP and GI. This aligns with earlier studies identifying seed vigor as a quantitatively inherited trait regulated by polygenes. Interestingly, three of the QTLs identified in this study overlap with loci previously mapped by other researchers. For example, *qSV*-*3* and *qSV*-*4* co-locate with genes *OsNCED3* and *OsABA1*, which regulate ABA synthesis in seeds—an essential component of germination control (Agrawal et al., 2001; Chen et al., 2023). *qSV*-*9* co-locates with *OsCNL2*, which is involved in SA biosynthesis and promotes submerged germination via OsGH3-mediated indole-acetic acid catabolism (Wang et al., 2024b). However, three novel loci, including *qSV*-*1*, were identified, emphasizing the reliability of our experimental approach and its contributions to expanding knowledge in seed vigor genetics.

Seed germination traits are interrelated with other agronomic characteristics, including seed dormancy, grain size, and grain quality (Wang et al., 2024a, 2025a; Yang et al., 2024; Xu et al., 2025). High seed vigor increases germination speed, which is desirable for direct-seeding systems but also carries the risk of reduced seed dormancy, potentially leading to pre-harvest sprouting during grain maturation. Several QTLs identified in this study, such as *qSV*-*3* and *qSV*-*4*, overlap with genes involved in ABA synthesis (*OsNCED3* and *OsABA1*), which promotes dormancy; mutations in these genes can lower ABA accumulation, enhancing germination but increasing the sprouting risk. Conversely, novel loci such as *qSV*-*1* and *qSV*-*8* may regulate seed vigor independently of the ABA and dormancy pathways. This opens up opportunities for genetic improvement strategies that balance seed vigor and dormancy through selective allele exploitation in breeding programs. By identifying haplotypes that optimize this balance, germination vigor traits can be refined to ensure high seedling vigor without compromising grain quality during harvesting.

CSSL populations offer a unique advantage for genetic studies due to their ability to control background genetic noise, allowing the precise detection of QTLs with minor effects or environmental interactions. In this study, 11 CSSLs surpassed Nipponbare in seed vigor traits, confirming the suitability of CSSLs for identifying subtle phenotypic variations. Furthermore, the use of next-generation sequencing (NGS) facilitated high-resolution physical mapping, overcoming traditional constraints posed by labor- and time-intensive marker development. NGS-generated SNP data enabled the robust identification of substituted chromosome segments, enhancing both QTL mapping accuracy and efficiency in marker development for breeding programs.

The major locus *qSV*-*1*, delimited to an 80.5-kb region on chromosome 1, contains 20 annotated genes, with *C3H10* and *OsOFP3* emerging as promising candidates. C3H10, encoding a CCCH-type zinc finger protein, likely modulates transcription in response to phytohormones, balancing ABA-induced dormancy and GA-promoted mobilization of resources—a vital axis for seed germination. Its expression patterns suggest a regulatory role in seed development and germination traits, aligning with findings from other zinc finger proteins reported to enhance ABA responsiveness (Seong et al., 2020). Likewise, OsOFP3, a key repressor of brassinosteroid biosynthesis, may fine-tune seed development and vigor by modulating embryo growth and physiological readiness for germination. Its expression declines during seed development, aligning with its potential function in regulating metabolic pathways essential for early seedling emergence (Xiao et al., 2020). Both genes represent valuable starting points for elucidating the molecular mechanisms governing seed vigor. Functional validation studies, such as gene-specific knockouts or overexpression experiments, are required to confirm the causal roles of these candidate genes. Further exploration of haplotypes and allelic variation within these genes may identify genetic variants that optimize seed vigor across diverse rice germplasms.

This study successfully identified six QTLs associated with early seed germination, representing significant genetic advances in rice seed vigor improvement. Among them, *qSV*-*1* represents a significant breakthrough, offering actionable insights for breeding rice varieties with enhanced seed vigor suitable for direct-seeding systems. By coupling CSSL mapping with whole-genome resequencing, this work sets a methodological benchmark for dissecting complex traits, ensuring more precise candidate gene characterization. Future research will focus on validating candidate gene functions and translating findings into breeding strategies that balance germination vigor, dormancy, and agronomic performance.

## Data Availability

The original contributions presented in the study are included in the article/**Supplementary Material**. Further inquiries can be directed to the corresponding authors.

## Supplementary material

Supplementary Table 1 Detailed genotype phenotype information about CSSLs.

Supplementary Table 2 Correlation analysis between GP, GR and GI.

Supplementary Table 3 the information about qSV-1 candidate genes.
