# Supplementary material for: Mapping of quantitative trait loci for early seed germination using whole-genome resequencing chromosome segment substitution lines of aus Kasalath in the background of japonica Nipponbare
Source: Front Plant Sci. 2025 Oct 20;16:1691426. doi: 10.3389/fpls.2025.1691426 (PMC12580301; doi:10.3389/fpls.2025.1691426)
Supplement: Supplementary file 2 [file DataSheet1.pdf]

Os01g0738400_Nipponbare
MAYETSSDHQLAAAAEFLAALQVHLAGAEASSPTWGGRCAYDEDFMMYEFKVRRCPRSRA 60
Os01g0738400_Kasalath
MAYETSSDHQLAAAAEFLAALQVHLAGAEASSPTWGGRCAYDEDFMMYEFKVRRCPRSRA 60

****

Os01g0738400_Nipponbare
HEWTSCPYAHPGEAARRRDPSHVTYTGEPCPDFRVAARAACPRGSGCPFAHGTFETWLHP 120
Os01g0738400_Kasalath
HEWTSCPYAHPGEAARRRDPSHVTYTGEPCPDFRVAARAACPRGSGCPFAHGTFETWLHP 120

****

Os01g0738400_Nipponbare
SRYRTRPCRSGMLCARPVCFFAHNDKELRIVGDDAAAATPSPRSPFTTSEDSPPPSPMDM 180
Os01g0738400_Kasalath
SRYRTRPCRSGMLCARPVCFFAHNDKELRIVGDDAAAATPSPRSPFTTSEDSPPPSPMDM 180

****

Os01g0738400_Nipponbare
KQIVLAMQQMDARKATRSVAPKTDMLQQELEEDAPELGWVSDLLM* 225
Os01g0738400_Kasalath
RQIVLAMQQMDARKATRSVAPKTDMLQQELEEDAPELGWVSDLLM* 225
:*************************************
****

Os01g0732300_Nipponbare
MDGGGSGAGRKKLKHRLAAILSVFTRRAGDRKRRGEEGAAAPPPPPPPLAFPSYSRLGGA 60
Os01g0732300_Kasalath
MDGGGSGAGRKKLKHRLAAILSVFTRRAGDRKRRGEEGAAAPP-PPPPLAFPSYSRLGGA 59

*** ****************

Os01g0732300_Nipponbare
GGKKAAAGKHERRLSLSVPRPPPLVNITIDCAGRRSVDAADPSLLAPLDLDARKMERRLT 120
Os01g0732300_Kasalath
GGKKAAAGKHERRLSLSVPRPPPLVNITIDCAGRRSVDAADPSLLAPLDLDARKMERRLT 119

****

Os01g0732300_Nipponbare
GTGLPYETGEWEGRKCPPSTPFAAAPPLARWKERASVSSRRLSTHSSRRLMSSSSSDDEY 180
Os01g0732300_Kasalath
GTGLPYETGEWEGRKCPPSTPFAAAPPLARWKERASVSSRRLSTHSSRRLMSSSSSDDEY 179

****

Os01g0732300_Nipponbare

```
DEDSRNLFSSRSFSSDSSDFYNCPRKNTRARASVSGPCRAPPPSSRRGTSQSCRYSFELP    240
Os01g0732300_Kasalath
DEDSRNLFSSRSFSSDSSDFYNCPRKNTRARASVSGPCRAPPPSSRRGTSQSCRYSFELP    239

************************************************************

Os01g0732300_Nipponbare
RGSTASAATDGGFAVVKRSSDPYEDFRKSMEEMIAEGAACGGGDGDGELSAERLLETYLV    300
Os01g0732300_Kasalath
RGSTASAATDGGFAVVKRSSDPYEDFRKSMEEMIAEGAACGGGDGDGELSAERLLETYLV    299

************************************************************

Os01g0732300_Nipponbare      LNSPRHYPAILAAFADVRETLFP*   323
Os01g0732300_Kasalath        LNSPRHYPAILAAFADVRETLFP*   322
                             ************************



Os01g0738400_promoter_Nipponbare      ----------
TTCGTCATTCTGCAGCAATGAGACCGCGGATGGGGCAGGTAATGAACACA      50
Os01g0738400_promoter_Kasalath
TGGGGTCGCCGCCGCCACTCGGGGCGGAGGAGAGAGGCGGC-----GTGAGATGGGAGGG    55
                                                      ** ** ** *     *
****  *  *         *  ***

Os01g0738400_promoter_Nipponbare
CCCAAATTCATTTATGAACTAATGACACCCAAAGACCAAAATGTGATGTTTCCTTTGCAT    110
Os01g0738400_promoter_Kasalath        AGGAAA-----
TGGGCTAGGGTTCGCGCCGAGGGAGTGGGTTTTGTTTCCACTATTGACG       110
                                          ***     *     *    *  * **
*  **       * ** *    *  ***

Os01g0738400_promoter_Nipponbare      CTCTCAGGTGGTT-
AGAGCACTAGATAGTTTAGCAGACTCTAACTTGAACAATGGCCTTC  169
Os01g0738400_promoter_Kasalath        CTCGCCGTCCGATCAAAACCCTAGAGA--
TCGGACGGCCGGTTCGTCGGCCGAATCGCCC 168
                                      *** * *   * * * * * ***** *  *
*  * *     * *   *     *   *

Os01g0738400_promoter_Nipponbare
AACCTGGCCGCAGTGAAGTATTCTTAGAACCACAGTCCGAAGAAATTAGATTGTTCCAGC    229
Os01g0738400_promoter_Kasalath
AAGAGGGATCGAGTGAGGCCTCTTT----------TCGGCCCAAGCCAGGTTGCGGCC--    216
                                      **   **    ***** *  *  **
** *   **   ** ***   *

Os01g0738400_promoter_Nipponbare
TGAGAGAATTCGGCAGCCGAGATTGTAGTGACGAGATGAGC-CAAGCTAGCTGGAGAAGC    288
Os01g0738400_promoter_Kasalath
TGGAGGAAAACTGCGCCAGCGCATGCAAAGGCGTGGGCCGACCGGCATAGGTCGGAAATA    276
                                      **   ***  * **  * * *  ** *  *
** *    *  *    *** * *  **
```

```
Os01g0738400_promoter_Nipponbare      CGAAGAGATTTGTAAACA--
TGTTCAGCACAAGATCATTAACAA---TTTTTTGGCCATT        343
Os01g0738400_promoter_Kasalath
AGTCTATTGTTGGTCCCTCGAGCCGAAAAAAAGAGAATAAGTACATTTTTAGTGCTCATT    336
                                       *   *   ***    *    *   *  *
****  ** *  *    ***  **  ****

Os01g0738400_promoter_Nipponbare      C----------
TTTACATGTAATTATCTCTTTTACATGATTTTTTTCCCCTC------TG       387
Os01g0738400_promoter_Kasalath
TTTTCAGAAGCATTACAATTATAGTTCTATTGAATATGTTTATTTCGAAGCAAACCTATA    396
                                                  *****  **    ***
**  * *** ** ***             *

Os01g0738400_promoter_Nipponbare      AGGCAAT-TCATGTTTC----
TCCATGGAATAAAATTTTGCATTC-------TTTGAGGA 435
Os01g0738400_promoter_Kasalath
AGAAAGTATTTTCTTTCTTTTTCCAATTAAATAATTGTTAAATGATGTTTGTATTAATAA    456
                                      **  * * *  * ****    ****   **
** * **  **          ** *  *

Os01g0738400_promoter_Nipponbare
GATAAGTAAATCATTCCTTCCCTTTTCAGTGAAGAGTATACAGATGTT---TGATTCTTG    492
Os01g0738400_promoter_Kasalath
AATTAGCAATTAAGCTCTGAAAATTCCAATA-AAATTCCAAAGAGCGTAACTAATTATGG    515
                                       ** ** ** * *   **     ** ** *
* * *  * ***   *   * *** * *

Os01g0738400_promoter_Nipponbare
GCATTCGCCGATAAACAACTGTATTGTTTTCGCTTCTTTTTGAGCAGGCCAGATTGCTGT    552
Os01g0738400_promoter_Kasalath
AGAATTTAATAAAATTAAATTTAACCATGTCA--TTTTATTTAACCATGGAGAATGGAGA    573
                                        * *     * **  ** * **    *
**   * ** ** * *     *** **  *

Os01g0738400_promoter_Nipponbare
CCGTGCACTGCAAGTCTCTGCGGGGATAAG--------------GCTGTAAAGCTTATCA    598
Os01g0738400_promoter_Kasalath
ATTTTACTTGTAAATTTATTTAATTATACACATACCTACTTAAAAATACCAAATATTTCA    633
                                         *    ** ** * * *      ***
*   **   * ***

Os01g0738400_promoter_Nipponbare
GTGGTTGCTTACATTCTATCCTCATGCCTTGTTTTGAGCACAAGTACAATCATTAGCGCC    658
Os01g0738400_promoter_Kasalath
GCGAATTTGTATTTTAATTAATTGCATATTTTTCTGATAGA-ACTTCCGTGCAACGCACG    692
                                      * *  *   **  **   *  *      **
** ***     * * *  *     ** *

Os01g0738400_promoter_Nipponbare
ACATTTCCGCAATGTTCGAATTGCTCTGCATCTTATTACAGTGTTCGCTTGCCGTAAGAG    718
Os01g0738400_promoter_Kasalath        GCACTTTGCTAAACCA-------
AACAACACTTTTGGTCAATATACACACTCTGTAATCA   745
                                       ** **    **            *  **
```

```
**    ** * * * *   * ****

Os01g0738400_promoter_Nipponbare
CAGGCCTGCTATTAGTCAGCTATAAACATATTTTAATAAGATAAAAAATGAGAGAGAAGA    778
Os01g0738400_promoter_Kasalath        CC-----------
CGCACGCGACGCACTTTTGCTAGTGAGATAAAA--------ATGAGA 786
                                      *             *   ** *   ** *
*  ** * ********        *  ***

Os01g0738400_promoter_Nipponbare
GCAGCGGGCTACAGATCTGTAGCTAGCTGTAGCACAGACTCCAAGACGCAATGTGTGTAT    838
Os01g0738400_promoter_Kasalath
GAAGAAGCGTGCAGATCAGAAAGTAGTAG--AAGACTGACTCAAGATACAATGTGTGTAT    844
                                      * **  *  * ****** * *  ***  *
*****  ************

Os01g0738400_promoter_Nipponbare      GATAGGTGAGACTATATATTAA-
TATATAGTAAGCAACTATTGTATAAATTAGCTATTAG   897
Os01g0738400_promoter_Kasalath
GATAGGTGGGACTATACATTAATAGTATAGTAAACAACTATTGTATAAATTGGCTATTAG    904
                                      ******** ******* *****
******** ***************** ********

Os01g0738400_promoter_Nipponbare
ATCGGCTATAAATGAATTGGAGCTAGTAGTAGGCTATACTGTTAAACTTCCTCTAACGGT    957
Os01g0738400_promoter_Kasalath        ATCGGCTATAAATGAATTGG---------
AGGGCTTTACTGTTAAACTTCCTCTAACGGT 955
                                      ********************
**** ************************

Os01g0738400_promoter_Nipponbare
GGTTGCCGCGTCACTGGCGACGCAAACACCTGCTGCTTAATACGTAGATCATTGAGATTC    1017
Os01g0738400_promoter_Kasalath
GGTTGCCGCGTCACTGGCGACGCAAACACCTGCTGCTTAATACGTAGATCATTGAGATTC    1015

************************************************************

Os01g0738400_promoter_Nipponbare
GCGTGGATCTCGCCTCTGTGTCGACCGATCGGCCGTTGATATGATATATAGCGGCGACCA    1077
Os01g0738400_promoter_Kasalath
GCGTGGATCTCGCCTCTGTGTCGACCGATCGGCCGTTGATATGATATATAGCGGCGACCA    1075

************************************************************

Os01g0738400_promoter_Nipponbare
CATGATGGGGGAGAAAACCCAGTGGGCGGATGGCAATGTCGCACCAAGAAATACGCGCAC    1137
Os01g0738400_promoter_Kasalath
CATGATGGGGGAGAAAACCCAGTGGGCGGATGGCAATGTCGCACCAAGAAATACGCGCAC    1135

************************************************************

Os01g0738400_promoter_Nipponbare
GAGCTGGTTGGCCTGGATTGATGGCGAGGCGAGCGCGGATGGACGTCGTCGCGTCGATCT    1197
Os01g0738400_promoter_Kasalath
```

GAGCTGGTTGGCCTGGATTGATGGCGAGGCGAGCGCGGATGGACGTCGTCACGTCGATCT   1195

** *********

Os01g0738400_promoter_Nipponbare
GTCGGTTGCCGTCGCCGTCTCCCTTTAATTTGCCTCCCCTTCTTTGCTTCGCCATGCCTG   1257
Os01g0738400_promoter_Kasalath
GTCGGTTGCCGTCGCCGTCTCCCTTTAATTTGCCTCCCCTTCTTTGCTTCGCCATGCCTG   1255

****

Os01g0738400_promoter_Nipponbare
TTCCTGGCCGTGTTTGCTGGGCCAAATCGAGATCGAAAGTTGGAAACCAGGGCCGCAGTA   1317
Os01g0738400_promoter_Kasalath
TTCCTGGCCGTGTTTGCTGGGCCAAATCGAGATCGAAAGTTGGAAACCAGGGCCGCAGTA   1315

****

Os01g0738400_promoter_Nipponbare
TATTGTTGGCCCAAGCCCAGCAAACCAGAGAAATACGGATCATACGGTTGCGTGTCCTGT   1377
Os01g0738400_promoter_Kasalath
TATTGTTGGCCCAAGCCCAGCAAACCAGAGAAATACGGATCATACGGTTGCGTGTCCTGT   1375

****

Os01g0738400_promoter_Nipponbare
ACATGCGTCGGCAATATGTGAAATCCTTGGGGGATAATAGTATTATTATTATCTCTCGGC   1437
Os01g0738400_promoter_Kasalath
ACATGCGTCGGCAATATGTGAAATCCTTGGGGGATAATAGTATTATTATTATCTCTCGGC   1435

****

Os01g0738400_promoter_Nipponbare
AGACCTACGATCTTACGTAGCTTACCTTAAATAATTGTTCGTGCTTCTGGCGTAAAGTAT   1497
Os01g0738400_promoter_Kasalath
AGACCTACGATCTTACGTAGCTTACCTTAAATAATTGTTCGTGCTTCTGGCGTAAAGTAT   1495

****

Os01g0738400_promoter_Nipponbare
TGAAGTGTGCAATAATAATCCGACTGCAGCTTGATCTGCTCGTATCTTTTAATAGCCGGC   1557
Os01g0738400_promoter_Kasalath
TGAAGTGTGCAATAATAATCCGACTGCAGCTTGATCTGCTCGTATCTTTTAATAGCCGGC   1555

****

Os01g0738400_promoter_Nipponbare
CGTCGCCGTGGCGACCATCGTCATCGCTAGTTCCCTAAAATTAACAGCTTGGCAGCATGA   1617
Os01g0738400_promoter_Kasalath
CGTCGCCGTGGCGACCATCGTCATCGCTAGTTCCCTAAAATTAACAGCTTGGCAGCATGA   1615

****

Os01g0738400_promoter_Nipponbare

TAAATTGATAATAGTTTACTCGTTGATTAATCCAATTCGTAATACATGGAGGAGTAAAGC 1677
Os01g0738400_promoter_Kasalath
TAAATTGATAATAGTTTACTCGTTGATTAATCCAATTCGTAATACATGGAGGAGTAAAGC 1675

****

Os01g0738400_promoter_Nipponbare
AGTTCGCTTCCGCAGCCCGCTGCGCTGCCAGGAACACCGTCGAGATCGCGAGACAGCTCC 1737
Os01g0738400_promoter_Kasalath
AGTTCGCTTCCGCAGCCCGCTGCGCTGCCAGGAACACCGTCGAGATCGCGAGACAGCTCC 1735

****

Os01g0738400_promoter_Nipponbare
CGAACGCAAAGCACCCACCCGCGGGCGATCGGGACGTGTCGCGCAGCA--TGCAAGCCGC 1795
Os01g0738400_promoter_Kasalath
CGAACGCAAAGCACCCACCCGCGGGCGATCGGGACGTGTCGCGCAGCATATGCAAGCCGC 1795

** **********

Os01g0738400_promoter_Nipponbare
CGCACCCGAGCCCTTCCACGTGCCCCAAGCTTCCGTCCCGGTACGTCCGTGTCCGCCCGG 1855
Os01g0738400_promoter_Kasalath
CACACCCGAGCCCTTCCACGTGCCCCAAGCTTCCGTCCCGGTACGTCCGTGTCCGCCCGG 1855
*
****

Os01g0738400_promoter_Nipponbare
GACAACCGACCCAGGACCGGACCCAACTCGAATACGGACTCGTGTCACTTCTTGCTTATA 1915
Os01g0738400_promoter_Kasalath
GACAACCGACCCAGGACCGGACCCAACTCGAATACGAACTCGTGTCACTTCTTGCTTATA 1915

************************************ ***********************

Os01g0738400_promoter_Nipponbare
AAAGGCAACGCATCGCCGCGCGCTCGCTCATCAAACAACATATAGCCGCGTCTCCTCATC 1975
Os01g0738400_promoter_Kasalath
AAAGGCAACGCATCGCCGCGCGCTCGCTCATCAAACAACATATAGCCGCGTCTCCTCATC 1975

****

Os01g0738400_promoter_Nipponbare TAATCCAACGCCACCGTGATCATCG 2000
Os01g0738400_promoter_Kasalath TAATCCAACGCCGCCGTGATCATCG 2000
************ ************

Os01g0732300_promoter_Nipponbare -----
TGAGGCAGCACCCCCTTGGATCATGACGAGCAGTGTCACCAATCAAGGCCGAGAC 55
Os01g0732300_promoter_Kasalath
TATTTTGAGGCAGCACCCCCTTGGATCATGACGAGCAGTGTCACCAATCAAGGCCGAGAC 60

****

```
Os01g0732300_promoter_Nipponbare
GAGATACTTCTGCACATGTCTCAGCTCAGGAACTATTGGCTCAAGCATTAAGATGAAATA    115
Os01g0732300_promoter_Kasalath
GAGATACTTCTGCACATGTCTCAGCTCAGGAACTATTGGCTCAAGCATTAAGATGAAATA    120

************************************************************

Os01g0732300_promoter_Nipponbare
AAAATACTAATACTAGAACTCAAGCAGCAGCAACGCATGCAAACCCTGTTGCTCATTGAT    175
Os01g0732300_promoter_Kasalath
AAAATACTAATACTAGAACTCAAGCAGCAGCAACGCATGCAAACCCTGTTGCTCATTGAT    180

************************************************************

Os01g0732300_promoter_Nipponbare
GCCTCGGCAATTCAGCACCTTTGCAATCAGGCCTTCACCAACTGACTTGCCTTTTGAGCT    235
Os01g0732300_promoter_Kasalath
GCCTCGGCAATTCAGCACCTTTGCAATCAGGCCTTCACCAACTGACTTGCCTTTTGAGCT    240

************************************************************

Os01g0732300_promoter_Nipponbare
GTTGATTGTAAACTATTGCCTCTGTACATGATTACTCAATCAACAGATTGGAGAATTGGG    295
Os01g0732300_promoter_Kasalath
GTTGATTGTAAACTATTGCCTCTGTACATGATTACTCAATCAACAGATTGGAGAATTGGG    300

************************************************************

Os01g0732300_promoter_Nipponbare
GAATACAAACAAGCTGCTACATCAACTACCCTAGGTTTTCTATTTGTATATCGTACAATC    355
Os01g0732300_promoter_Kasalath
GAATACAAACAAGCTGCTACATCAACTACCCTAGGTTTTCTATTTGTATATCGTACAATC    360

************************************************************

Os01g0732300_promoter_Nipponbare
TCAGCTTTTTCTGTTTGTTGTTTTCTGATTATAAGCTGGAGCATTCTGTTGGTTAAAAAC    415
Os01g0732300_promoter_Kasalath
TCAGCTTTTTCTGTTTGTTGTTTTCTGATTATAAGCTGGAGCATTCTGTTGGTTAAAAAC    420

************************************************************

Os01g0732300_promoter_Nipponbare
AACTGCTATCAGCTGGTCATGATTGGGAATCCCAGTTTTTGTTTGTTATGTGCTCCCTTG    475
Os01g0732300_promoter_Kasalath
AACTGCTATCAGCTGGTCATGATTGGGAATCCCAGTTTTTGTTTGTTATGTGCTCCCTTG    480

************************************************************

Os01g0732300_promoter_Nipponbare
GACCCTGCATCACACACATGGCAAGGAGTACATGCATGATGCATTTGACATGTTAAAGAA    535
Os01g0732300_promoter_Kasalath
GACCCTGCATCACACACATGGCAAGGAGTACATGCATGATGCATTTGACATGTTAAAGA-    539
```

****

Os01g0732300_promoter_Nipponbare
TGATTTGTGTGATGTACAGAGTATTGAAAATGCCATATTATTGGGATATATTATACAATG 595
Os01g0732300_promoter_Kasalath
AGATTTGTGTGATGTACAGAGTATTGAAAATGCCATATTATTGGGATATATTATACAATG 599

****

Os01g0732300_promoter_Nipponbare
TTTTCTTTTATCTTAAGGTTCAAGACCTTTCAAGGAACGGTTTCTGTGATGGGCAGAATG 655
Os01g0732300_promoter_Kasalath
TTTTCTTTTATCTTAAGGTTCAAGACCTTTCAAGGAACGGTTTCTGTGATTGGCAGAATG 659

** *********

Os01g0732300_promoter_Nipponbare
CATGGAACAGGCCACATTATTGGGATCCATCATACAATGGTTTCTTTTATCACATGCTTA 715
Os01g0732300_promoter_Kasalath
CATGGAACAGGCCACATTATTGGGATCCATCATACAATGGTTTCTTTTATCACATGCTTA 719

****

Os01g0732300_promoter_Nipponbare
ATCAAGTGCGTGCTGGCACAAGTCCGCCTGGAGAATGGAGATAGAAAAATGCTCAGGTAA 775
Os01g0732300_promoter_Kasalath
ATCAAGTGCGTGCTGGCACAAGTCCGCCTGGAGAATGGAGATAGAAAAATGCTCAGGTAA 779

****

Os01g0732300_promoter_Nipponbare
TTGCAGGCTAGTTTGATAGTACTAGTAAACTGTTCTTATTAGTAGGATCCCATGCTGATG 835
Os01g0732300_promoter_Kasalath
TTGCAGGCTAGTTTGATAGTACTAGTAAACTGTTCTTATTAGTAGGATCCCATGCTGATG 839

****

Os01g0732300_promoter_Nipponbare
TGTGTTTCGTTTGTTAGGCCTCTCTCCTTTCTTAGGTTTGCCCTTCCCATCTGTCCTCAG 895
Os01g0732300_promoter_Kasalath
TGTGTTTCGTTTGTTAGGCCTCTCTCCTTTCTTAGGTTTGCCCTTCCCATCTGTCCTCAG 899

****

Os01g0732300_promoter_Nipponbare
TTCTGTCTTACTGCTAACAAAATACTTAACACCTGTATGTTTGTCTTTATAAGGTCAGAT 955
Os01g0732300_promoter_Kasalath
TTCTGTCTTACTGCTAACAAAATACTTAACACCTGTATGTTTGTCTTTATAAGGTCAGAT 959

****

Os01g0732300_promoter_Nipponbare
CTTAAAAGTCGCGCGCCGTCTACGTGATAGATATAGTAGTTAGTACTATAGTAGATAGAC 1015

Os01g0732300_promoter_Kasalath
CTTAAAAGTCGCGCGCCGTCTACGTGATAGATATAGTAGTTAGTACTATAGTAGATAGAC 1019

****

Os01g0732300_promoter_Nipponbare
ACTATTGTGCTTATTAAACCCCAAAAGAACAGCTCAGCTGGCTCCCACTCGCACAGCTGG 1075
Os01g0732300_promoter_Kasalath
ACTATTGTGCTTATTAAACCCCAAAAGAACAGCTCAGCTGGCTCCCACTCGCACAGCTGG 1079

****

Os01g0732300_promoter_Nipponbare
AGGACGCACTTGGCACTAAACAACGCATTAGGGGCAAATCGACTTCTAATGCCACGAAGA 1135
Os01g0732300_promoter_Kasalath
AGGATGCACTTGGCACTAAACAACGCATTAGGGGCAAATCGACTTCTAATGCCACGAAGA 1139
****
****

Os01g0732300_promoter_Nipponbare
AGCTGCATTCGACAAATTGACAAAGTGAAGCTTATACATGGTTCTGTTCAACGCTTCAAT 1195
Os01g0732300_promoter_Kasalath
AGCTGCATTCGACAAATTGACAAAGTGAAGCTTATACATGGTTCTGTTCAACGCTTCAAT 1199

****

Os01g0732300_promoter_Nipponbare
GAACGTGTTGATTGCTTCGTAAAAGTAAAACTACACTAGCATGTACTATTGGTACCGTCC 1255
Os01g0732300_promoter_Kasalath
GAACGTGTTGATTGCTTCGTAAAAGTAAAACTACACTAGCATGTACTATTGGTACCGTCC 1259

****

Os01g0732300_promoter_Nipponbare
AGTGGTGTACAGGCTACAAACTGAAGAGTTTGCCGTGGTCAACACTAGCACATGCCCGGC 1315
Os01g0732300_promoter_Kasalath
AGTGGTGTACAGGCTACAAACTGAAGAGTTTGCCGTGGTCAACACTAGCACATGCCCGGC 1319

****

Os01g0732300_promoter_Nipponbare
CGGCCGACTGTACAGTAAAACTGGGGGGCAAGTTGCAGCCTTGCAGTCTTGCAGAGAGCA 1375
Os01g0732300_promoter_Kasalath
CGGCCGACTATACAGTAAAACTGGGGGGCAAGTTGCAGCCTTGCAGTCTTGCAGAGAGCA 1379
****
****

Os01g0732300_promoter_Nipponbare
GTTCACGCTCAGAAAGAATGTATGCCAAGTCGCCAACCCGCCCACAATTCCTTCTCCCTT 1435
Os01g0732300_promoter_Kasalath
GTTCACGCTCAGAAAGAATGTATGCCAAGTCGCCAACCCGCCCACAATTCCTTCTCCCTT 1439

****

```
Os01g0732300_promoter_Nipponbare
TCCCTTTCCATCTCCTCTCCCAATTTGTGTCCCAAAACTGCACCAACACTGCAACTCACC    1495
Os01g0732300_promoter_Kasalath
TCCCTTTCCATCTCCTCTCCCAATTTGTGTCCCAAAACTGCACCAACACTGCAACTCACC    1499

************************************************************

Os01g0732300_promoter_Nipponbare
TTGTTCAGCATCAGTTCACAAGCTGCCCTATGGATATGATCCTTTGTTATCCACGTGCAC    1555
Os01g0732300_promoter_Kasalath
TTGTTCAGCATCAGTTCACAAGCTGCCCTATGGATATGATCCTTTGTTATCCACGTGCAC    1559

************************************************************

Os01g0732300_promoter_Nipponbare
GCAATATCACGAGCCACCCCCCAGCAGAAGCCAACATTACCGCAAGATAATATAAGCCGA    1615
Os01g0732300_promoter_Kasalath
GCAATATCACGAGCCACCCCCCAGCAGAAGCCAACATTACCGCAAGATAATATAAGCCGA    1619

************************************************************

Os01g0732300_promoter_Nipponbare
AACATCCAGCCAGGAGAGGCCGGGGCCGGGTCGCAGCCAGTCACGCAGCAGAGCGCGTAA    1675
Os01g0732300_promoter_Kasalath
AACATCCAGCCAGGAGAGGCCGGGGCCGGGTCGCAGCCAGTCACGCAGCAGAGCGCGTAA    1679

************************************************************

Os01g0732300_promoter_Nipponbare
CCGCCGCCTCGGGCCATTTCTCACACAAACTCCCACTACGTTACATGTATCCTGTCACGG    1735
Os01g0732300_promoter_Kasalath
CCGCCGCCTCGGGCCATTTCTCTCACAAACTCCCACTACGTTACATGTATCCTGTCACGG    1739
                                      **********************
*************************************

Os01g0732300_promoter_Nipponbare
TCAGCCACGTCGCGGAGGGCAGTGCGACCGAGCGAAGCGAGCGCCCACGTCCCCGCGCAC    1795
Os01g0732300_promoter_Kasalath
TCAGCCACGTCGCGGAGGGCAGTGCGACCGAGCGAAGCGAGCGCCCACGTCCCCGCGCAC    1799

************************************************************

Os01g0732300_promoter_Nipponbare
TCTGGACTGGCGGTTTTTGTTCTGTAGCTAGCGGCTAGCGCTTGTGCCACAACCAGTTTA    1855
Os01g0732300_promoter_Kasalath
TCTGGACTGGCGGTTTTTGTTCTGTAGCTAGCGGCTAGCGCTTGTGCCGCAACCAGTTTA    1859

************************************************ ***********

Os01g0732300_promoter_Nipponbare
AAGCCCCGCGAGAGGGCGCGCATTACCCCTCCTGGACGAATGAGATAGACCAACCCCCCA    1915
Os01g0732300_promoter_Kasalath
AAGCCCCGCGAGAGGGCGCGCATTACCCCTCCTGGACGAATGAGATAGACCAACCCCCCA    1919
```

```
************************************************************

Os01g0732300_promoter_Nipponbare
CTCCCGCGCGCTCATCCATCCATCCATCCATCCATCCATACACGCAGCGCACGAACAAGA    1975
Os01g0732300_promoter_Kasalath
CTCCCGCGCGCTCATCCATCTATCCATCC----ATCCATACACGCAGCGCACGAACAAGA    1975
                                      ******************** ********
***************************

Os01g0732300_promoter_Nipponbare      CAGAGTCACGGGCACGAAGTCACTC 2000
Os01g0732300_promoter_Kasalath        CAGAGTCACGGGCACGAAGTCACTC 2000
                                      *************************
```
